# Characterization of different CTC subpopulations in non-small cell lung cancer

**DOI:** 10.1038/srep28010

**Published:** 2016-06-15

**Authors:** Annkathrin Hanssen, Jenny Wagner, Tobias M. Gorges, Aline Taenzer, Faik G. Uzunoglu, Christiane Driemel, Nikolas H. Stoecklein, Wolfram T. Knoefel, Sebastian Angenendt, Siegfried Hauch, Djordje Atanackovic, Sonja Loges, Sabine Riethdorf, Klaus Pantel, Harriet Wikman

**Affiliations:** 1 Department of Tumour Biology, University Medical Centre Hamburg-Eppendorf, Hamburg, Germany; 2 QIAGEN Hannover GmbH, Germany; 3 Center for Neurology, Neurosurgery, and Psychiatry, Department of Psychiatry, Campus Benjamin Franklin, Charité University Hospital Berlin, Germany; 4 Department of General, Visceral and Thoracic Surgery, University Medical Centre Hamburg-Eppendorf, Hamburg, Germany; 5 Department of General, Visceral and Pediatric Surgery, University Hospital and Medical Faculty of the Heinrich-Heine University Düsseldorf, Düsseldorf, Germany; 6 Department of Internal Medicine II and Clinic (Oncology Centre), University Medical Centre Hamburg-Eppendorf, Hamburg, Germany

## Abstract

Circulating tumour cells (CTCs) serve as valuable biomarkers. However, EpCAM positive CTCs are less frequently detected in NSCLC patients compared to other epithelial tumours. First, EpCAM protein expression was analysed in primary and metastatic lung cancer tissue. In both groups 21% of the samples were EpCAM negative. Second, the CellSearch system identified 15% of patients (n = 48) as CTC positive whereas a multiplex RT-PCR for *PIK3CA, AKT2, TWIST,* and *ALDH1* following EGFR, HER2 and EpCAM based enrichment detected CTCs in 29% of the patients. Interestingly, 86% of CTC positive patients were found to express ALDH1. Only 11% of the patients were CTC-positive by both techniques. CTC positivity was associated with patient disease state when assessed by the multiplex RT-PCR assay (p = 0.015). Patients harbouring tumours with an altered *EGFR* genotype were more frequently CTC-positive compared to patients with EGFR wildtype tumours. In subsets of patients, CTCs were found to express genes involved in resistance to therapy such as *HER3* and *MET*. In conclusion, using multiple targets for CTC capture and identification increases the sensitivity of CTC detection in NSCLC patients, which can be explained by the presence of different CTC subtypes with distinct molecular features.

Lung cancer is the most common cause of cancer-related mortality worldwide. For 2015, 1.4 million deaths were predicted with increasing incidence rates among women in Europe^1^. Even after complete primary tumour resection, about 45% of early stage non-small cell lung cancer (NSCLC) patients develop local or distant recurrence within 8 to 18 months^2^. These distant metastases originate from single migratory tumour cells that, detached from the primary tumour, survive in the circulation and finally colonize distant target tissue. Numerous studies showed that circulating tumour cells (CTCs) are able to predict metastatic relapse and are associated with disease progression and worse clinical outcome. Therefore, CTCs have been added to the TNM staging system in 2010 for breast cancer (cM0 (i+)) and have great potential as disease monitoring tool during treatment of cancer patients^3^,^4^. At present, the CellSearch system is the only FDA-cleared CTC detection method available on the market and is based on the isolation of EpCAM positive cells^5^. However, due to several reasons, including epithelial-to-mesenchymal transition (EMT), CTCs have been shown to downregulate epithelial proteins such as EpCAM^6^,^7^. These EMT-associated CTCs could, therefore be missed by EpCAM-based enrichment technologies^6^. In NSCLC, CellSearch generally detects less CTCs compared to other tumour entities^8^. Positivity rates of 33–36% were reported in patients with metastatic disease^9^. Patients with earlier disease stages were either CTC negative (stage IIIA) or rarely positive (4%, stage IIIB)^10^. Although infrequent, CTC detection by CellSearch was found to be significantly associated with shorter overall survival, indicating that also in NSCLC EpCAM positive cells have prognostic value^11^. During the last years, numerous studies were published using different approaches to isolate and detect CTCs in NSCLC^9^. CTC detection rates varied between 50–100% but not all studies found a significant prognostic and/or predictive value. However, a meta-analysis clearly demonstrated a clinical impact of these CTCs underlining the fact that different CTC subpopulations can be of predictive value^11^.

In this study, we assessed the frequency of EpCAM expression in both primary tumours and metastatic tissue of NSCLC patients. In addition, we explored the frequency of CTCs using the CellSearch system and the novel Adna-EMT-2 test to study the appearance of another CTC subpopulation besides the strictly EpCAM-positive CTCs in NSCLC and investigated the molecular properties of these cells.

## Results

### EpCAM protein expression in primary and metastatic lung cancer tissue

**Figure 1:**
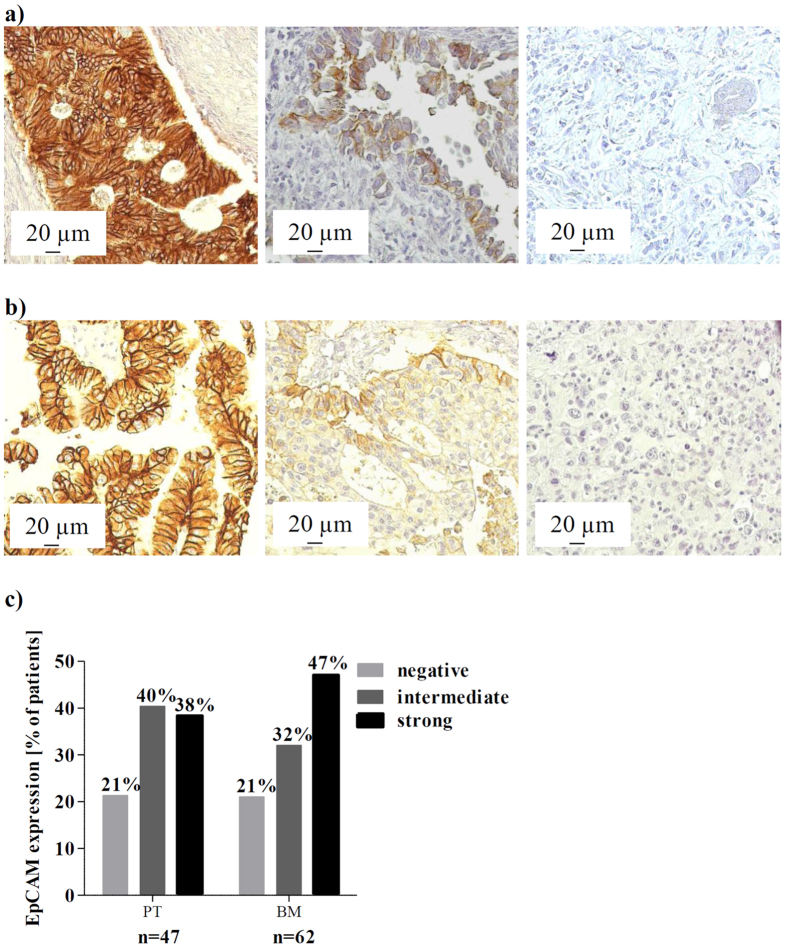
EpCAM protein expression and distribution in primary and metastatic lung cancer tissue. EpCAM IHC staining was performed on tissue microarrays from (**a**) primary NSCLC tumour tissue (PT; n = 47) and from (**b**) lung cancer brain metastases (BM; n = 62). Representative tissues of a strong (left), intermediate (centre) and negative (right) staining are shown (200x magnified, Axioplan 2 Imaging, Zeiss). (**c**) EpCAM frequency distribution in primary lung and brain metastatic tissue. EpCAM expression was analysed and quantified by microscopic analysis of two independent scientists.

In order to analyse whether EpCAM might get lost during NSCLC cancer progression, EpCAM protein expression was investigated in primary lung tumours (n = 55) as well as brain metastases (n = 76) by IHC. Evaluable results were obtained from 47 and 62 patients, respectively. Tumour samples were classified into negative, intermediate, and strongly positive and representative stainings are shown in Fig. 1a,b. In total, 78.7% of primary tumour tissue samples were EpCAM positive, with 40.4% showing intermediate and 38.3% evidencing strong EpCAM expression (Fig. 1c). Out of all primary tumours, 21.3% were EpCAM negative. Concerning the brain metastatic tissue samples, 79.0% were EpCAM positive, with 32.3% showing intermediate and 46.7% showing strong EpCAM expression. 20.9% of patients’ brain metastases were EpCAM negative. In addition, nine corresponding primary tumours and brain metastases were analysed for EpCAM expression. Here we observed a constant expression in 56% of patients (n = 9). In two patients, EpCAM expression was downregulated and two other patients showed an upregulation of EpCAM expression. EpCAM expression in primary tumour tissue was not significantly associated with any clinicopathological factors (Supplementary Table 2). Kaplan-Meier analysis did not reveal any association with survival (data not shown).

### CTC detection in NSCLC

To study the appearance of another CTC subpopulation in NSCLC besides the EpCAM-positive CTCs, peripheral blood from 48 patients was collected and analysed using the CellSearch system and the multiplex RT-PCR (mRT-PCR) assay. The specificity and sensitivity of the mRT-PCR assay were confirmed prior to the start of patient recruitment (96% specificity; 92% sensitivity for *PIK3CA, AKT2* and *TWIST* and 94% for *ALDH1*, data not shown). A blood sample was defined as CTC positive if at least one CTC was detected by CellSearch or one of the target genes was expressed above threshold concentration^12^. Patients were grouped according to their disease and treatment state: M0 resectable NSCLC (n = 17), M1 chemo-naïve disease (n = 9), M1 chemo-treated progressive disease (n = 10), M1 chemo-treated non-progressive disease (n = 12).

**Figure 2:**
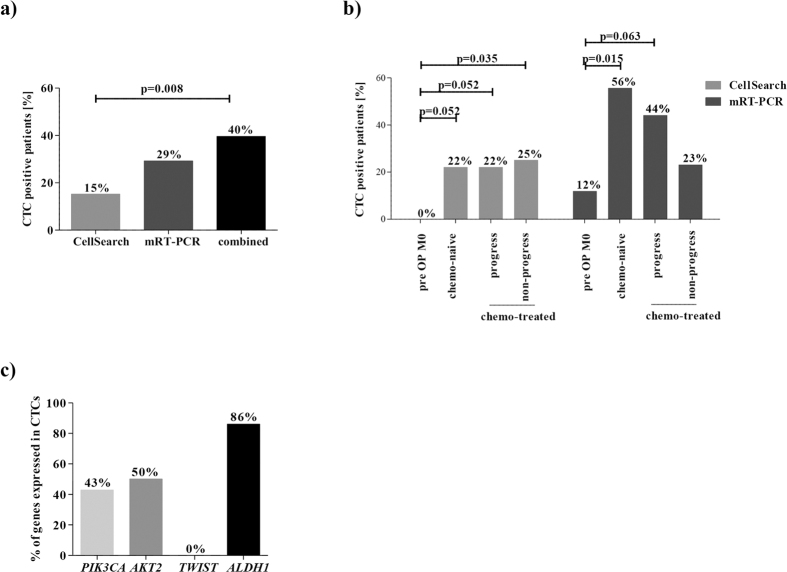
NSCLC CTC positivity rates detected by CellSearch and Adna mRT-PCR test. (**a**) The mRT-PCR detected CTCs in 29% and CellSearch in 15% of NSCLC patients (n = 48). The combined CTC status revealed a significantly increased CTC burden compared to CellSearch alone (40% versus 15%, p = 0.008). (**b**) CTC positivity rates in different groups of patients. The cohort was grouped into operated patients M0 (n = 17), chemo-naïve (n = 9) and -treated patients M1, the latter either in a progressive (n = 10) or non-progressive (n = 12) state. (**c**) Gene expression on Adna-EMT-2 isolated CTCs. *PIK3CA, AKT2, TWIST* and *ALDH1* gene expression were measured by mRT-PCR.

Using the CellSearch system, 15.2% (n = 7) of all patients had ≥1 CTC per 7.5 ml of blood. Of these seven positive patients, five had ≥3 CTCs/7.5 ml (10.9%). The mean number of CTCs detected by the CellSearch system was 6.3 per 7.5 ml blood (range 1–13). 29.2% (n = 14) of the patients were classified as CTC positive with the mRT-PCR (Fig. 2a). When CellSearch and mRT-PCR CTC positivity were combined, significantly more patients were found to be CTC positive compared to when the CellSearch method alone was used (39.6%, n = 19; p = 0.008).

Using CellSearch, none of the non-metastatic patients was positive for CTCs prior to primary tumour resection. In contrast, 22.2–25.0% of metastatic patients that were either chemo-naïve or -treated including progressive and non-progressive disease were positive for CTCs (Fig. 2b). CTC detection was significantly associated with a non-progressive M1 disease status when compared to M0 patients (p = 0.035). Furthermore, a borderline association was found between CTC positivity in M1 chemo-naïve or progressive disease and M0 patients (p = 0.052).

The mRT-PCR revealed a positivity rate for CTCs in 11.8% of resected M0 patients (Fig. 2b). Chemo-naïve metastatic patients were positive in 55.6% of the cases, in line with 44.4% of patients with progressive disease. Only in 23.1% of patients with a non-progressive M1 disease state, CTCs were found. Hereby, CTC positivity was significantly associated with a chemo-naïve metastatic setting compared to the non-metastatic group (p = 0.015). Furthermore, a borderline association of CTC positivity was detected between patients under disease progress and non-metastatic patients (p = 0.063). In conclusion, the mRT-PCR seems to be more sensitive in NSCLC than the CellSearch, and the CTC detection rate was depending on the patient’s disease state.

Further analysis revealed a low concordance rate between both methods. Only 10.5% (n = 2) of the positive cases gave a positive result by both techniques, whereas 63.2% were solely positive with the mRT-PCR and 26.3% were solely positive by CellSearch. This indicates the presence of different CTC subpopulations in NSCLC patients.

Concerning the mRT-PCR positive patients, the target genes were found to be heterogeneously expressed in CTCs and among the different patient groups (Fig. 2c). The stem cell marker *ALDH1* was found to be the most prominent expressed gene with an expression rate of 85.7%, whereas *AKT2* and *PIK3CA* were found in 50.0% and 42.9% of the tumour cells, respectively. The mesenchymal marker *TWIST* was not detected in any CTCs. Furthermore, *PIK3CA* and *ALDH1* positive CTCs were found to be significantly more frequent in patients with a chemo-naïve or progressive metastatic disease compared to M0 and non-progressive M1 patients (*PIK3CA*: p = 0.010; *ALDH1*: chemo-naïve: p = 0.003; progress: 0.017), whereas *AKT2* positive CTCs were found in all groups (Supplementary Figure 1). After Ficoll density gradient centrifugation, two potential cancer stem cells that express ALDH1 but were negative for CD45 were detected (Supplementary Figure 2).

### CTC characterization based on therapy-resistance related gene expression in NSCLC

In a subset of 22 patients, CTCs were detected by additional semi-quantitative RT-PCR (RT-qPCR) analysis after the Adna-EMT-2 based CTC enrichment step. The following NSCLC therapy related genes were chosen: *ERCC1, MET, HER3, JAG1* and *VIM.* Raw data (Cq-values) from patient samples are provided in the supplementary data section (Supplementary Table 5). The RT-qPCR detection results highly resembled those of the mRT-PCR (Table 1). At least one of the target genes were positive with the RT-qPCR panel in 85.7% (n = 7) of the mRT-PCR positive cases (Table 1), indicating that the technique itself is limited by the enrichment and not the detection step. *HER3* was found to be the most prominent gene expressed in mRT-PCR positive cases (85.7%), followed by *VIM* (71.4%), *JAG1* (57.1.4%), *ERCC1* (57.1%) and *MET* (14.3%). When taking the whole cohort into account, *MET* was detected at a high frequency (75.0%) in M0 resectable NSCLC but not in M1 (7.1%) patients.

### CTC positivity and the relation to patient clinical characteristics

**Figure 3:**
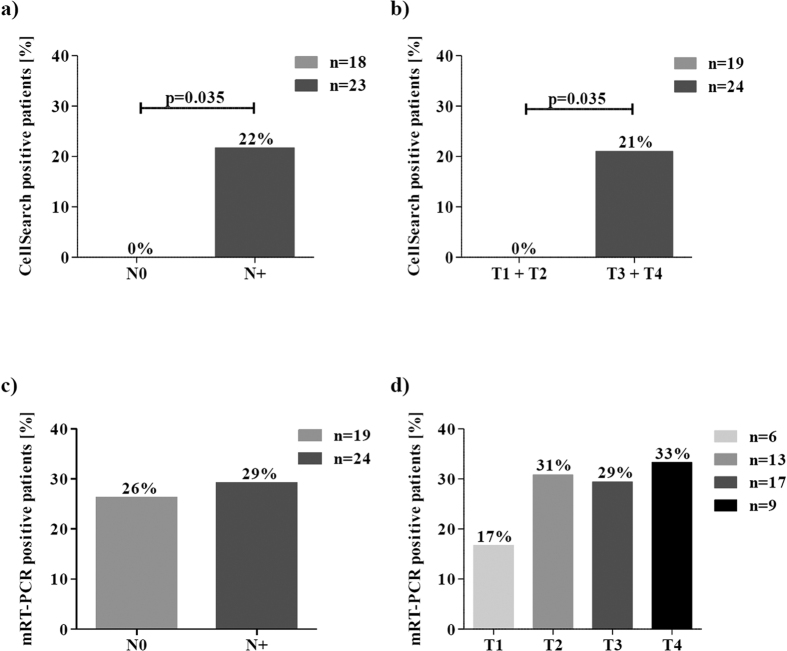
Association between CTC positivity and clinicopathological parameters. (**a,b**) CellSearch CTC positivity and (**c,d**) mRT-PCR CTC positivity in relation with the presence of lymph node metastases and the size of the primary tumour.

The CellSearch system identified CTCs only in patients with lymph node metastases (21.7%; p = 0.035) and larger primary tumours (≥T3) (20.8%, p = 0.035) (Fig. 3a,b). In contrast, the mRT-PCR detected CTCs at almost the same frequency in both, lymph node positive and negative patients (29.2% versus 26.3%, Fig. 3c). Furthermore, the mRT-PCR revealed 16.7% of T1 tumour patients and 29.4–33.3% of T ≥ 2 patients as CTC-positive (Fig. 3d), indicating that CTC detection based on the mRT-PCR identifies CTCs independently from the TNM status (p = 0.836).

**Figure 4:**
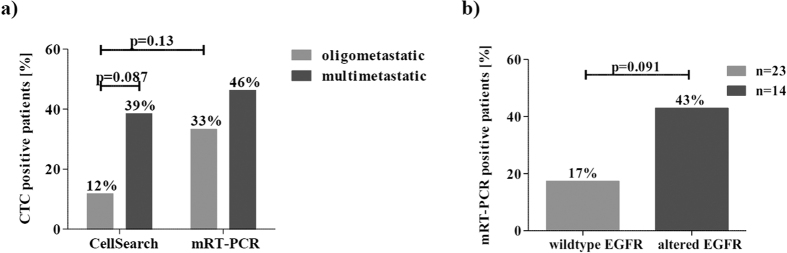
CTC positivity in patients with multiple metastases and altered EGFR primary tumours. (**a**) CellSearch versus mRT-PCR CTC positivity. The mRT-PCR detects more CTC positive patients in a single metastatic disease setting. (**b**) mRT-PCR CTC positivity in relation to EGFR genotype: Patients with an altered EGFR genotype in the primary tumour including EGFR mutations, amplification, and polysomy are more frequently CTC positive compared to wildtype primary tumours when using the mRT-PCR.

Patients with only one metastatic site were differentiated from patients with multiple metastases. With CellSearch, only 11.8% of single-metastatic patients had detectable CTCs, whereas 38.5% of multi-metastatic patients were CTC positive (p = 0.087). With the mRT-PCR, only slightly less CTC positive patients were found in the single-metastatic group compared to multi-metastatic patients (33.3% versus 46.2%) (Fig. 4a). The mRT-PCR test tended to detect more CTCs (42.9%) among patients with an altered EGFR status of the primary tumour, including EGFR mutations, amplifications and high polysomy compared to patients with an EGFR wildtype status (17.4%, p = 0.091) (Fig. 4b).

Survival analyses were, however, not performed as the survival status was only available for 22 patients and the patient blood was drawn at different treatment time points. CTC positivity and patient survival status is reported in Supplementary Table 6.

## Discussion

The FDA-approved CellSearch CTC detection system isolates CTCs based on their EpCAM expression. It has been shown in various epithelial tumour entities that metastatic patients with CTCs have a significantly decreased progression-free and overall survival^5^. Also in NSCLC, CellSearch detected CTCs are associated with worse patient outcome^11^. However, only around 35% of metastatic patients are CTC positive^9^. Previous studies revealed a higher CTC incidence when using EpCAM-independent enrichment techniques^9^. Therefore, our hypothesis was that in NSCLC a significant number of CTCs remain undetected by EpCAM-based techniques.

In a first step, we analysed EpCAM expression in primary lung tumours as well as brain metastases, in order to investigate whether EpCAM might get lost during NSCLC cancer progression. According to the IHC analysis, 21.3% of the primary tumours were negative for EpCAM and only 38.3% strongly expressed EpCAM, which is in line with earlier reports^13^. The expression of EpCAM in brain metastases was similar to that of the primary tumours, indicating that EpCAM does not seem to be permanently downregulated in metastases to a larger extent. Furthermore, a rather small cohort of corresponding primary tumors and metastatic tissues revealed that EpCAM expression may vary to some extent. Thus, the EpCAM status of tumour tissue does not necessarily predict the EpCAM status in CTCs. Nevertheless, a loss of EpCAM expression in one fifth of primary and metastatic tissue and the apparently transient expression of the protein indicates that another CTC subpopulation could exist in NSCLC, which potentially remains undetected by EpCAM-based methods. We therefore tested a new CTC detection method in addition to the CellSearch system.

In our study, CellSearch did not detect CTCs in non-metastatic patients, but identified 22.2–25.0% of CTC positive patients upon metastatic disease setting, thereby resembling the classical view of the CellSearch CTC impact^10^,^14^,^15^. Furthermore, our positivity rate for CTCs was associated with a positive lymph node status as previously reported^11^. Also in early stage patients, our CellSearch analysis was based on a total blood volume of 7.5 ml. Krebs *et al*. described earlier that stage I and stage II patients are CTC-negative when using this method^10^. We therefore conclude that a higher volume of blood is necessary to assess a potential clinical significance of CTCs in early stage NSCLC patients. In general, the mRT-PCR assay detected CTCs at higher frequencies compared to CellSearch (29.2% versus 15.2%, respectively). The test showed that especially metastatic patients with either a chemo-naïve or a progressive diseases status were more frequently CTC positive compared to non-metastatic patients or non-progressive patients. These results indicate that the CTC status assessed by the mRT-PCR might reflect the clinical stage of the patients. It would be worth to evaluate whether the mRT-PCR test could be a tool for monitoring response to treatment by serial blood samplings during the treatment. In addition, CTCs could be detected in 42.9% of patients with an altered EGFR status in the primary tumour. Importantly, this positivity rate was not influenced by the patient disease state. Although EGFR represents a clinically very important target in NSCLC, the occurrence of treatment resistance hampers clinical success^16^. Zhang *et al*. described EGFR +/HER2+/HPSE+/Notch1+ cells without EpCAM expression as highly aggressive CTCs which are competent to colonize the brain as metastatic site^17^. These highly aggressive but EpCAM-negative CTCs are not captured by the CellSearch system but might play an important role. Patients with an altered EGFR status might potentially harbour more highly aggressive CTCs. Interestingly, in 50% of patients the EGFR mutation is accompanied by a gene amplification of the other allele^18^. For these patients, the Adna EMT-2 test should be optimal, as it is based on the isolation of EGFR expressing CTCs. At present, tumour characteristics are analysed by operation or biopsy. The less invasive liquid biopsy offers the opportunity to elaborate the patient disease state repetitively and therefore allows real-time monitoring of the disease^19^. Furthermore, therapeutic targets such as activating EGFR mutations have been detected on CTCs and could thus be used to predict personalized therapies^4^,^20^. Our mRT-PCR test could serve as targeted diagnostic tool to monitor EGFR TKI treatment response.

Another interesting aspect is that 85.7% of our CTCs detected by mRT-PCR were positive for *ALDH1*, indicating that in NSCLC a large fraction of CTCs are of stemness character. ALDH1 positive lung cancer cells were previously shown to have unique stem cell characteristics, including initiation of tumorigenesis, multipotent differentiation potential and resistance to chemotherapy^21^. Moreover, the expression of ALDH1 in NSCLC primary tumours has been associated with shorter overall survival in early stage NSCLC (stage I)^21^,^22^. Remarkably, breast cancer CTCs that express the stem cell marker *ALDH1* have been suggested to be involved in metastatic disease progression, with one study showing that 76% of CTC positive patients carried ALHD1 positive CTCs^23^,^24^. ALDH1 is thus of interest as a metastasis initiating factor expressed in CTCs. Others have shown that CTCs in breast cancer, which express stem cell factors are able to initiate metastatic outgrowth^25^,^26^,^27^. Furthermore, EGFR is discussed to be involved in the process of EMT and drug resistance and is also expressed on the metastatic initiating CTCs^28^,^29^. We thus think that ALDH1 positive CTCs are isolated when using our CTC enrichment cocktail including EGFR, indicating that also in NSCLC CTCs seem to have stem cell characteristics.

In a subset of patients, we additionally assessed the expression level of five genes potentially involved in therapy resistance mechanisms by RT-qPCRs. EGFR mutated tumours treated with tyrosine kinase inhibitors (TKI) may become resistant by different means^30^,^31^. Most commonly, a secondary mutation T790M can outcompete the TKIs^32^. This mutation has been detectable in CTCs of EGFR resistant NSCLC patients^20^. Alternatively, overexpression of MET protein or HER3 have been described in TKI resistant tumours^31^. Importantly, our results show that also the therapy-resistance related genes *MET* and *HER3* can be detected on CTC level and thus could be of use to monitor therapy resistance. In this exploratory study, most patients did not receive TKI. However, one patient received EGFR TKI and developed a resistance after 7 months of treatment. According to our RT-qPCR findings, this patient harboured HER3 positive CTCs, which is described as one of the major EGFR TKI resistance mechanisms^30^. A new study is needed to substantiate this interesting observation. In particular, *MET* was often detected in patients with M0 resectable NSCLC classified as mRT-PCR negative. A recent study postulated that CTCs that can form tumours in mice express MET as a metastases-initiating factor^27^. The clinical validation needs to be performed in future studies.

## Conclusion

In this study, we could show that the usage of multiple cell surface proteins for CTC isolation in NSCLC increases sensitivity for CTC identification. CTC positivity based on the mRT-PCR was strongly depending on tumour burden, enabling the possibility to serve as a disease and therapy resistance monitoring test, especially perhaps among EGFR positive NSCLC patients. Despite the promising results of the present investigation, the clinical validity needs to be assessed in larger studies on NSCLC patients.

## Methods

### Patient cohort

A total number of 48 NSCLC patients from the University Medical Center Hamburg Eppendorf (UKE) and the University Medical Centre Düsseldorf in Germany were analysed for CTCs. The experimental protocol was approved (Approval No. PVN-3779) by the Ethics Committee of the Chambers of Physicians of the State of Hamburg. The methods were carried out in accordance with the approved guidelines and patients were enrolled between June 2013 and June 2015. In addition, blood samples were analysed for CTCs from patients of the University Medical Centre Düsseldorf. The samples were taken between November 2014 and June 2015 according to protocol #4664 approved by the Ethics Committee of the Medical Faculty of the Heinrich-Heine-University Düsseldorf. All participants provided written informed consent or were tested for CTC in the context of tumour staging (included to TNM staging in 2010). Blood was drawn into adequate collection tubes and processed within 24 hours (Adna-EMT-2 test) or 24–76 hours (CellSearch). Patients were recruited according to the following criteria: early stage (M0) resectable NSCLC patients, chemo-naïve patients with distant metastases and chemo-treated patients with distant metastases either in a clinically progressive or non-progressive state. The non-progressive patients’ group includes both patients in a stable disease state and patients in remission. Tissue microarrays (TMA) with primary lung tumours (n = 55) and brain metastases (n = 76), resected at UKE in Hamburg, were used for EpCAM expression analysis. For each patient, tissue was analysed in duplicates with a core size of 1.0 mm^33^. All patient characteristics are reported in supplementary Table 1.

### Staining of primary and metastatic lung cancer tissue by immunohistochemistry (IHC)

To verify the EpCAM expression, two TMAs consisting of 55 primary lung tumour tissues and 76 lung cancer brain metastases were stained for EpCAM by IHC. In addition, the tissue from nine matching primary tumour and brain metastasis samples was analysed. The tissue slides were de-paraffinized with xylol and rehydrated with an ethanol series followed by a 10 min trypsin digestion step (37 °C, Sigma-Aldrich). Unspecific binding was blocked using DAKO ChemMate peroxidase blocking solution (Dako, Denmark) and the tissue was incubated with the primary EpCAM antibody (1:75, NCL Esa antibody; Novocastra, UK) for 60 min at room temperature. For visualization, the DAKO Envision kit was used with DAKO DAB solution (Dako Denmark A/S, Glostrup, Denmark) and hemalaun (Merck, Germany) as counterstain. The tissue was analysed blinded for signal intensity by two independent researchers (AH, HW). Both signal intensity and signal distribution were multiplied to a final value and grouped according to a threshold of ≤0.5 into negative, ≤2 into intermediate and ≥2 into strong stainings.

### CTC isolation and detection by CellSearch

For CellSearch analysis, 7.5 ml of blood was drawn into CellSave tubes (Janssen Diagnostics, LLC) and processed within 76 hours. The semi-automated analysis was performed as described elsewhere^34^. In short, EpCAM coated ferro-fluids were used to isolate CTCs followed by a cytokeratin expression and DAPI based identification step and an automated scanning process. CD45 immunofluorescence staining was used to identify unspecifically enriched leukocyte fractions. Cells were evaluated by an experienced scientist (SR).

### CTC detection by Adna-EMT-2 isolation and mRT-PCR-based detection

For Adna EMT-2 test, 5 ml of blood was drawn into Adna Collect tubes (AdnaGen, Germany), and processed within 24 hours. Briefly, this technique is based on an immuno-magnetic bead enrichment step targeting the surface proteins EpCAM, HER2 and EGFR. Isolated target cells are then lysed and mRNA is reversely transcribed into cDNA followed by a mRT-PCR. For CTC detection targeting survival genes (*PIK3CA* and *AKT2*), the EMT marker gene *TWIST* and the stem cell marker gene *ALDH1.* The threshold for gene expression originates from the manufacturer’s instructions for the use of the AdnaTest EMT2/StemCell (QIAGEN Hannover) and was developed and is quality controlled in frequently repeated healthy donor blood experiments concerning cell enrichment as well as target gene profiling. We additionally assessed the target gene expression in 50 healthy donors to confirm the specificity of the cut-off value given from the manufacturer. Furthermore, spiking experiments with 30 tumour cells (MiaPaca: *PIK3CA, AKT2* and *TWIST*; PC3: *ALDH1*) were performed to also confirm the sensitivity of our technique. This technique is here named as mRT-PCR assay. PCR products were detected on an Agilent 2100 Bioanalyser (Agilent Technologies, USA). Samples were classified as positive when at least one of the marker genes was expressed above threshold concentration of ≥0.25 ng/μl (*ALDH1*: ≥0.15 ng/μl). *ACTIN* was used as internal PCR control.

### CTC detection by Adna-EMT-2 isolation and RT-qPCR-based detection

For the RT-qPCR analyses, the Adna-EMT-2 cDNA was used for gene specific pre-amplification with the TATAA Multiplex Grand Master Mix according to the manufacturer’s instructions (QIAGEN Hannover GmbH). PCR activation was performed at 95 °C for 3 minutes and followed by 16 cycles of denaturation at 95 °C for 20 seconds, extension for 20 seconds at 60 °C and elongation at 72 °C for 3 minutes, respectively. Singleplex RT-qPCR was performed for *Excision Repair Cross-Complementation Group 1* (*ERCC1*)*, Erb-B2 Receptor Tyrosine Kinase 3* (*HER3*)*, MET proto oncogene* (*MET*)*, Jagged 1* (*JAG1*), and *VIMENTIN* (*VIM*) as well as for the control genes *CD45* (leucocyte control) and *GAPDH* (housekeeping gene) using iTaq Universal Supermix SYBR Green Mix™ (Biorad, Hercules, CA, USA). RT-qPCR was performed using the StepOnePlus™ (Life Technologies, Carlsbad, CA, USA) real time system. After PCR activation at 95 °C for 3 minutes, the thermal profile of 35 cycles in total was as follows: 20 seconds at 95 °C, 20 seconds at 60 °C, 30 seconds at 72 °C and 1 minute at 95 °C. Additionally, melting curves were performed. For data analysis GAPDH was used to qualify a sample in a sense that, if not detectable, the sample would not qualify. It was assumed that several genes of interest are not exclusively expressed in CTCs. Therefore, a cut-off value was calculated for each gene separately in a way that the false positive rate in all HDs (n = 28) was lower than 10% (specificity > = 90%). We provide all Cq values from healthy donors in the supplementary data section Table 3. Using these gene specific cut-off values delta Cq was calculated as ∆Cq = Cq_cut-off_ − Cq_sample_. Healthy donor samples confirmed a specificity of 100% for *ERCC1* and *JAG1* and 96% for *VIM*, *MET* and *HER3* (Supplementary data Table 4). This detection assay is named as RT-qPCR and was used to analyse a subset of 22 patients. Patient disease status and CTC expression results are listed in Table 1.

### Ficoll centrifugation and CTC detection based on immunofluorescence staining

CTCs are isolated according to their physical properties by Ficoll density gradient centrifugation. In short, whole blood is layered on Ficoll and then centrifuged for 30 min at room temperature. Ery-lysis is done for 3 min and isolated peripheral mononuclear blood cells are cyto-centrifuged onto glass slides (500000 cells per slide)^35^. For detection of CTCs, immunofluorescence stainings were performed using the pan-cytokeratin antibody (1:80; AE1AE3, eBioScience) and ALDH1 antibody (1:100; ab52492, Abcam) in combination with a species-specific secondary antibody (1:200; anti-rabbit-Alexa488, LifeTechnologies), and a CD45 antibody (1:150; Clone HI30, BioLegend). Cells were fixed with 0.5% para-formaldehyde and blocked with AB serum. All antibodies were incubated for 45 min at room temperature except for ALDH1 (overnight, 4 °C). Glass slides were covered and manually analysed by fluorescence microscopy.

### Statistical analysis

The chi square, two-tailed Fischer’s exact test or two-tailed T-test were used to identify group differences and potential associations between the investigated variables and the clinical and histopathological risk factors using either the SPSS software package, version 21.0 (SPSS Inc. Chicago, IL) or Microsoft Office Excel (2013). A p-value of <0.05 was considered as statistically significant. All p-values are two-sided. Kaplan-Meier in line with the long-rank test was used to analyse survival differences between the groups.

## Additional Information

**How to cite this article**: Hanssen, A. *et al*. Characterization of different CTC subpopulations in non-small cell lung cancer. *Sci. Rep.* **6**, 28010; doi: 10.1038/srep28010 (2016).

## Supplementary Material

Supplementary Information

## Figures and Tables

**Table 1:** Therapy-resistance related gene expression on CTCs: Additional RT-qPCR with the target genes *ERCC1, MET, HER3, JAG1* and *VIM* was performed and CTC positivity was compared to mRT-PCR results.

Patient	Group	Treatment	Adna- EMT2	*ERCC1*	*MET*	*HER3*	*JAG1*	*VIM*
1	M0	–	pos	neg	neg	neg	neg	neg
2	M0	–	neg	neg	pos	neg	neg	neg
3	M0	–	neg	neg	pos	neg	pos	neg
4	M0	–	neg	neg	pos	neg	neg	neg
5	M0	–	neg	neg	neg	neg	neg	neg
6	M0	–	neg	neg	pos	neg	neg	neg
7	M0	–	neg	neg	pos	neg	neg	neg
8	M0	–	neg	neg	pos	neg	neg	neg
9	pre-chemo	–	pos	pos	pos	pos	pos	pos
10	pre-chemo	–	pos	pos	neg	pos	pos	pos
11	progress	chemotherapy	pos	pos	neg	pos	pos	pos
12	progress	chemotherapy	pos	pos	neg	pos	pos	pos
13	progress	chemotherapy	pos	neg	neg	neg	neg	pos
14	progress	TKI, chemotherapy	pos	neg	neg	pos	neg	neg
15	progress	chemotherapy	neg	neg	neg	neg	neg	neg
16	progress	chemotherapy	neg	neg	neg	neg	neg	neg
17	progress	chemotherapy	neg	neg	neg	neg	neg	neg
18	progress	chemotherapy	neg	neg	neg	neg	neg	neg
19	non-progress	chemotherapy	neg	neg	neg	neg	neg	neg
20	non-progress	chemotherapy	neg	neg	neg	neg	neg	neg
21	non-progress	chemotherapy	neg	neg	neg	neg	neg	neg
22	non-progress	chemotherapy	neg	neg	neg	neg	neg	neg

85.7% of mRT-PCR positive cases were also identified positive by the RT-qPCR.
