# Supplementary material for: Characterization of different CTC subpopulations in non-small cell lung cancer
Source: Sci Rep. 2016 Jun 15;6:28010. doi: 10.1038/srep28010 (PMC4908396; doi:10.1038/srep28010)
Supplement: Supplementary Information [file srep28010-s1.pdf]

Supplementary information

Characterization of different CTC subpopulations in non-small cell lung cancer

Annkathrin Hanssen[1], Jenny Wagner[2], Tobias M. Gorges[1], Aline Taenzer[1,3], Faik G. Uzunoglu[4], Christiane Driemel[5], Nikolas H. Stoecklein[5], Wolfram T. Knoefel[5], Sebastian Angenendt[5,6], Siegfried Hauch[2], Djordje Atanackovic[7], Sonja Loges[1,7], Sabine Riethdorf[1], Klaus Pantel[1], Harriet Wikman[1*]

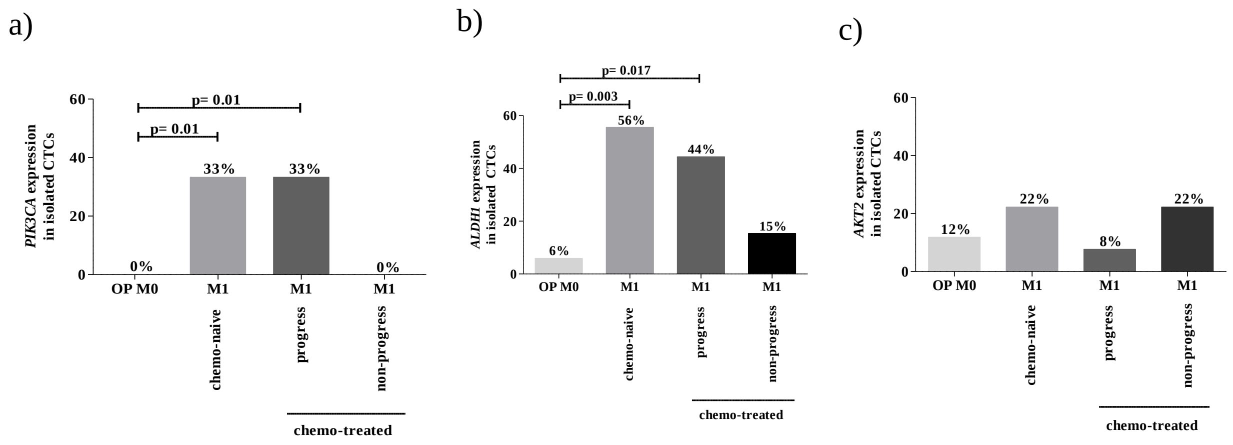

Supplementary Figure 1: Relation of patient disease state with gene expression of Adna-EMT-2 isolated CTCs. a) *PIK3CA* (p=0.01) and b) *ALDH1* (p=0.017) positive CTCs were significantly associated with a chemo-naïve (n=9) or a progressive disease (n=9) state. c) *AKT2* positive CTCs were detected in patients at all disease states (M0 patients: n=17; non-progress patients: n=13).

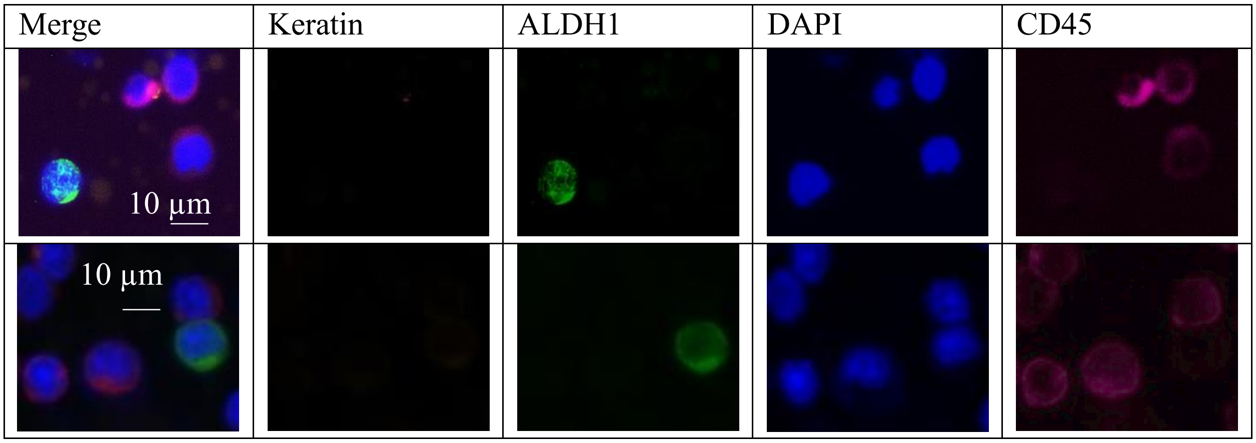

Supplementary Figure 2: Potential detection of CTCs with a stemness character. CTCs were isolated based on Ficoll centrifugation and stained for ALDH1, keratin and CD45 expression. Two cells were detected in a subset of six advanced NSCLC patients.

Supplementary Table 1: Clinical characteristics of NSCLC patients recruited for CTC analysis.

Clinical characteristics			n	%
Histology	AC		32	67
	SCC		14	29
	n.a.		2	4
Gender	Female		20	42
	Male		28	58
Tumor size at time of first diagnosis				
	T1		6	13
	T2		13	27
	T3		17	35
	T4		9	19
	n.a.		3	6
Lymph Node status at time of first diagnosis				
	N0		19	40
	N1+		24	50
	n.a.		5	10
Metastatic status at time of first diagnosis				
	M0		20	42
	M1		16	33
	n.a.		2	25
Metastatic disease at time of enrollment				
	Single-metastatic		18	38
	Poly-metastatic		13	27
	n.a.		17	35
UICC stage	I		3	6
	II		5	10
	III		6	13
	IV		13	27
	n.a.		20	44
Primary tumor operated		yes	28	58
		no	20	42
Treatment at time of enrollment				
	chemo-naïve	yes	28	58
		no	20	42
	radiotherapy	yes	12	25
		no	36	75
	EGFR TKI	yes	2	4
		no	46	96
Death	yes		15	31
	no		14	29
	n.a.		19	40

Supplementary Table 2: Clinical characteristics of primary tumor patients on tissue microarray and percentage of tissues with negative, intermediate and strong EpCAM expression.

Clinical characteristics		n	% negative	% intermediate	% strong	p-value
Histology						
	AC	22	24	52	24	0.162
	SCC	21	31	31	38	
	other	12	0	37.5	62.5	
Gender						
	Female	21	20	43	37	0.863
	Male	34	24	35	41	
Tumor size						
	T1	18	7	60	33	0.415
	T2	23	23.8	28.6	47.6	
	T3	7	33.3	33.3	33.3	
	T4	7	40	40	20	
Lymph Node status						
	N0	25	22	39	39	0.984
	N1+	30	20.8	41.7	37.5	
Metastatic status						
	M0	51	18.6	39.5	41.9	0.176
	M1	4	50	50	0	
Stage at diagnosis						
	I	17	20	40	40	0.525
	II	16	15	54	31	
	III	17	21	29	50	
	IV	4	50	50	0	
	n.a.	1				
Local relapse						
	yes	9	43	14	43	0.189
	no	41	16	43	41	
	n.a.	5				
Metastatic relapse						
	yes	10	0	14	86	0.054
	no	40	21.2	42.4	36.4	
	n.a.	5				
Smoking						
	yes	49	24	43	33	
	no	1	0	0	0	
	n.a.	5				
Death						
	yes	36	25	37.5	37.5	0.899
	no	18	19	42	39	
	n.a.	1				

Supplementary Table 3: Cut-off determination of the RT-qPCR analysis. Target gene expression was measured in 28 healthy donors to determine

Sample	Sex	Select Kit	*ERCC1*	*VIM*	*MET*	*HER3*	*JAG1*	*GAPDH*	*GAPDH* quality
1	F	EMT2	31.10	34.92	35.00	35.00	35.00	24.60	ok
2	F	EMT2	28.88	31.69	35.00	35.00	35.00	21.92	ok
3	F	EMT2	29.90	32.14	35.00	35.00	35.00	22.68	ok
4	F	EMT2	28.48	33.59	35.00	35.00	35.00	23.85	ok
5	F	EMT2	29.90	32.66	35.00	35.00	35.00	22.73	ok
6	F	EMT2	28.78	30.85	35.00	35.00	35.00	21.67	ok
7	F	EMT2	29.09	31.44	35.00	35.00	35.00	22.55	ok
8	F	EMT2	29.37	29.46	35.00	35.00	35.00	19.80	ok
9	M	EMT2	35.00	35.00	35.00	35.00	35.00	25.39	ok
10	M	EMT2	29.93	33.84	35.00	35.00	35.00	23.30	ok
11	M	EMT2	30.30	33.42	35.00	35.00	35.00	23.33	ok
12	M	EMT2	32.25	33.15	35.00	35.00	35.00	22.94	ok
13	F	EMT2	30.77	34.15	35.00	35.00	35.00	23.51	ok
14	F	EMT2	30.91	33.76	35.00	35.00	35.00	24.50	ok
15	M	EMT2	29.67	32.85	35.00	35.00	35.00	21.83	ok
16	M	EMT2	32.07	32.80	35.00	35.00	35.00	22.90	ok
17	F	EMT2	29.44	34.24	35.00	35.00	35.00	22.87	ok
18	F	EMT2	28.73	31.72	35.00	35.00	35.00	21.06	ok
19	M	EMT2	29.16	31.96	35.00	35.00	35.00	21.54	ok
20	M	EMT2	28.71	30.82	35.00	32.78	35.00	20.95	ok
21	F	EMT2	27.75	31.52	32.88	33.54	35.00	21.33	ok
22	F	EMT2	29.04	31.71	35.00	29.01	35.00	21.54	ok
23	F	EMT2	31.04	34.84	35.00	35.00	35.00	23.48	ok
24	F	EMT2	28.18	32.37	32.59	35.00	35.00	21.59	ok
25	n.a.	EMT2	28.05	31.92	35.00	35.00	35.00	21.28	ok
26	F	EMT2	30.24	32.65	32.52	34.85	35.00	21.92	ok
27	M	EMT2	27.75	30.41	27.78	35.00	35.00	18.65	ok
28	F	EMT2	26.99	30.88	35.00	35.00	35.00	20.47	ok
		average	29.69	32.53	34.49	34.65	35.00		
		2xSD	3.30	2.88	3.02	2.42	0.00		
		**Cut-off:**	**26.39**	**29.65**	**31.48**	**32.23**	**34.00**		

Supplementary Table 4: Specificity determination of the RT-qPCR analysis.

Sample	Sex	Select Kit	*ERCC1*	*VIM*	*MET*	*HER3*	*JAG1*
1	F	EMT2	-4.71	-5.27	-3.52	-2.77	-1.00
2	F	EMT2	-2.48	-2.04	-3.52	-2.77	-1.00
3	F	EMT2	-3.50	-2.49	-3.52	-2.77	-1.00
4	F	EMT2	-2.09	-3.94	-3.52	-2.77	-1.00
5	F	EMT2	-3.50	-3.01	-3.52	-2.77	-1.00
6	F	EMT2	-2.39	-1.20	-3.52	-2.77	-1.00
7	F	EMT2	-2.70	-1.79	-3.52	-2.77	-1.00
8	F	EMT2	-2.98	0.19	-3.52	-2.77	-1.00
9	M	EMT2	-8.61	-5.35	-3.52	-2.77	-1.00
10	M	EMT2	-3.54	-4.19	-3.52	-2.77	-1.00
11	M	EMT2	-3.91	-3.77	-3.52	-2.77	-1.00
12	M	EMT2	-5.86	-3.50	-3.52	-2.77	-1.00
13	F	EMT2	-4.37	-4.50	-3.52	-2.77	-1.00
14	F	EMT2	-4.52	-4.11	-3.52	-2.77	-1.00
15	M	EMT2	-3.27	-3.20	-3.52	-2.77	-1.00
16	M	EMT2	-5.68	-3.15	-3.52	-2.77	-1.00
17	F	EMT2	-3.04	-4.59	-3.52	-2.77	-1.00
18	F	EMT2	-2.34	-2.07	-3.52	-2.77	-1.00
19	M	EMT2	-2.77	-2.31	-3.52	-2.77	-1.00
20	M	EMT2	-2.32	-1.17	-3.52	-0.55	-1.00
21	F	EMT2	-1.35	-1.87	-1.40	-1.32	-1.00
22	F	EMT2	-2.64	-2.06	-3.52	3.22	-1.00
23	F	EMT2	-4.64	-5.19	-3.52	-2.77	-1.00
24	F	EMT2	-1.78	-2.72	-1.11	-2.77	-1.00
25	n.a.	EMT2	-1.66	-2.27	-3.52	-2.77	-1.00
26	F	EMT2	-3.85	-3.00	-1.04	-2.62	-1.00
27	M	EMT2	-1.35	-0.76	3.70	-2.77	-1.00
28	F	EMT2	-0.60	-1.23	-3.52	-2.77	-1.00
	**Specificity:**		**100%**	**96%**	**96%**	**96%**	**100%**

Supplementary Table 5: Raw data (Cq-values) from RT-qPCR analysis of patient samples.

Patient	*ERCC1*	*MET*	*HER3*	*JAG1*	*VIM*	*GAPDH*	*GAPDH* quality
1	30.55	32.95	34.92	35.00	35.00	24.00	ok
2	29.03	33.44	29.11	35.00	35.00	22.66	ok
3	29.15	33.06	29.20	35.00	33.22	22.72	ok
4	26.90	31.55	31.15	35.00	35.00	21.17	ok
5	29.57	35.00	31.52	35.00	35.00	24.85	ok
6	27.17	31.91	29.22	35.00	34.01	22.70	ok
7	28.75	32.36	30.84	35.00	35.00	22.84	ok
8	27.78	31.51	29.42	35.00	35.00	20.98	ok
9	23.93	24.86	26.92	25.08	27.55	15.09	ok
10	26.22	26.95	33.37	25.84	28.61	17.74	ok
11	22.77	23.56	31.78	27.93	27.95	13.91	ok
12	24.95	25.94	35.00	29.97	28.32	15.90	ok
13	28.11	28.42	33.76	35.00	35.00	18.89	ok
14	28.34	30.68	33.41	27.71	34.63	18.92	ok
15	32.79	32.34	34.02	36.64	36.67	23.02	ok
16	28.27	31.96	35.00	35.00	35.00	21.54	ok
17	29.04	31.01	34.52	35.00	34.28	21.92	ok
18	30.57	33.13	33.80	35.98	36.04	21.88	ok
19	29.09	30.73	35.00	35.00	35.00	20.22	ok
20	35.00	33.37	33.85	35.00	35.00	22.76	ok
21	30.22	32.21	35.00	35.00	35.00	21.87	ok
22	31.82	33.73	35.00	35.00	35.00	22.22	ok

Supplementary Table 6: Survival data, treatment intervention and CTC positivity of M1 patients.

Patient	Group	death	FUP (days)	Chemo therapy	Radia tion	targeted therapy	Cell Search ®	MRT -PCR
23	pre-chemo	yes	422	no	yes	no	neg	neg
24	pre-chemo	no	637	no	yes	no	neg	neg
25	pre-chemo	yes	184	no	no	no	neg	pos
26	pre-chemo	yes	32	no	yes	yes	neg	pos
27	pre-chemo	yes	183	no	no	no	neg	neg
28	pre-chemo	yes	223	no	yes	no	pos	pos
10	pre-chemo	yes	97	no	yes	no	neg	pos
29	non-progress	no	589	yes	no	no	neg	pos
19	non-progress	yes	240	yes	yes	no	neg	neg
20	non-progress	no	330	yes	yes	no	neg	neg
30	non-progress	yes	38	yes	no	no	pos	neg
31	non-progress	no	477	yes	yes	no	n.a.	neg
32	non-progress	yes	105	yes	yes	no	pos	neg
22	non-progress	yes	316	yes	yes	no	neg	neg
11	progress	yes	385	yes	yes	no	pos	pos
12	progress	no	840	yes	no	no	neg	pos
13	progress	yes	779	yes	no	yes	neg	pos
14	progress	yes	98	yes	no	yes	neg	pos
15	progress	no	790	yes	yes	no	pos	neg
33	progress	no	600	yes	no	no	neg	neg
17	progress	yes	222	yes	yes	yes	neg	neg
18	progress	yes	227	yes	no	no	neg	neg
